# Electronic lab notebooks: can they replace paper?

**DOI:** 10.1186/s13321-017-0221-3

**Published:** 2017-05-24

**Authors:** Samantha Kanza, Cerys Willoughby, Nicholas Gibbins, Richard Whitby, Jeremy Graham Frey, Jana Erjavec, Klemen Zupančič, Matjaž Hren, Katarina Kovač

**Affiliations:** 1 University of Southampton, Southampton, UK; 2 BioSistemika, Ljubljana, Slovenia

**Keywords:** Electronic lab notebooks (ELNs), Notebooking software, Cloud, Semantic web, Scientific software

## Abstract

Despite the increasingly digital nature of society there are some areas of research that remain firmly rooted in the past; in this case the laboratory notebook, the last remaining paper component of an experiment. Countless electronic laboratory notebooks (ELNs) have been created in an attempt to digitise record keeping processes in the lab, but none of them have become a ‘key player’ in the ELN market, due to the many adoption barriers that have been identified in previous research and further explored in the user studies presented here. The main issues identified are the cost of the current available ELNs, their ease of use (or lack of it) and their accessibility issues across different devices and operating systems. Evidence suggests that whilst scientists willingly make use of generic notebooking software, spreadsheets and other general office and scientific tools to aid their work, current ELNs are lacking in the required functionality to meet the needs of the researchers. In this paper we present our extensive research and user study results to propose an ELN built upon a pre-existing cloud notebook platform that makes use of accessible popular scientific software and semantic web technologies to help overcome the identified barriers to adoption.

**Electronic supplementary material:**

The online version of this article (doi:10.1186/s13321-017-0221-3) contains supplementary material, which is available to authorized users.

## Background

In scientific research, communication is essential; between researchers, funding bodies, industry, and members of the public. Ideas need to be shared, evidence disseminated, plans discussed, findings recorded, and errors corrected. Researchers may work alone, but their research is of little value to the scientific community if it isn’t disseminated. The scientific record can act as a legally binding record that protects intellectual property (IP) [39]. Historically the paper laboratory notebook and the scientific paper have been at the centre of this scientific communication [12]; however this is being slowly replaced by the arrival of digital technologies and the Internet and the Web in particular [7].

Digital Technologies are shaping the way experiments are performed, results captured, and findings disseminated. Computers enable a myriad of functions that benefit researchers/scientists, they can be searched, shared, easily backed up, and readily accessed [18]. They facilitate interactive computation, electronic communication, multimedia, and digital information management [50]. Within the lab, instruments are mostly computer controlled; computers are the main tools for capturing, analysing, and annotating data. Electronic laboratory notebooks (ELNs) are also transforming the way that the scientific record is captured with a revolutionary transformation from paper notebooks to the digital capture of experiments [6].

ELNs offer significant benefits to researchers by facilitating long-term storage, reproducibility, and enhanced availability of experiment records across multiple devices, ensuring standard operating procedure compliance and providing interfaces to instrumentation, supporting IP protection, collaboration, and open science [4, 21, 24, 43, 49]. ELNs eliminate the need for manual transcription and can be used by distributed groups [32], facilitate managing notes, and simplify the inclusion and curation of digital resources (e.g. instrument data, analysis results) [2]. While some systems are restricted to repositories of raw data and results, others have the potential to support researchers through the whole experiment lifecycle [17, 23].

More recently, semantic lab notebooks (SLNs) have utilised semantic web technologies to expose research data as formalised metadata [10], and to link between the different data sets collected throughout the experimental process [42]. Incorporating semantic web technologies within ELNs, using RDF and ontologies to enrich the data with meaning and context, provides new functionality such as making inferences about experiment types, and creates valuable links between experiment outcomes and their final reports [2, 5, 10, 23, 32]. Making ELN data machine readable increases interoperability, facilitates integration with third party tools and enables automatic generation of materials for deposition in an archive or publication [10], increasing the usefulness of the tools for researchers.

Although ELNs are being increasingly used for industrial research, uptake in academia is limited [19, 35]. This paper explores the current offerings of ELNs and Electronic Notebook software. Our research conducted studies to investigate the attitudes of academics towards ELNs, and their desired functionality. It presents an overview of the barriers to adoption within academic environments, researcher behaviour, and key features for ELNs. Following these findings, we discuss priorities for future ELN development and propose our Semantic Platform based ELN solution. Table 1 introduces the user studies that will be discussed in this paper.

**Table 1:** A table describing the different user studies that have been detailed in this paper

Study	Study dates	No of participants	Description
A—BioSistemika’s Webinar Survey [3]	Oct 2015 and Feb 2016	228	Survey of current ELN usage
B—BioSistemika’s ELN Survey	Mar–Apr 2015	196	Survey of ELN features, costs and barriers
C—University of Southampton’s ELN study	Summer 2016	103 ELNs	Study of the current ELN market: active/inactive ELNs, ELN licensing and platforms
D—University of Southampton lab practice study (focus groups and lab observations)	Nov 2016–Mar 2017	33	Focus groups with physicists, chemists and biologists. Lab observations of four different chemistry labs at the University to better understand current lab practice
E—University of Southampton’s Dial-a-Molecule (DaM) Survey and iLabber Pilot Project	Sep 2011	Initial Survey—88 Start of Trial—92 End of Trial—93	Surveys to gain knowledge and understand attitudes towards using ELNs and issues identified with using the trialled ELN
F—University of Southampton’s Communities Survey	2010–2015	94	Full details of this study can be found in [48]

## The market today

The current ELN Market is oversaturated with choice; however, despite the wide range of products available there is no obvious ‘leader’. Additionally, the scientific community is still resistant to using ELNs, despite the popularity of Electronic Notebooks. Electronic Notebooks that have been subverted to ELN usage, current ELN offerings, and the attitudes to ELNs and their current usage have been examined and detailed in this section.

### Electronic notebooks

Today’s market has multiple offerings for Electronic Notebooks: (Microsoft Word, Office 365, Google Docs), Evernote [16] and OneNote [30], which have been evaluated for use as ELNs [33, 46, 47]. Oleksik et al.’s [33] study reported that the collaborative features of OneNote facilitated faster and easier sharing, and enabled simultaneous communication between researchers, irrespective of location. Users trialling Evernote as an ELN [47] said they appreciated the electronic affordances such as ‘accessible from any online computer’ and ‘ability to search’, but found that it was lacking in domain knowledge; stating that it was ‘simple and practical for some laboratories, but for others it does not offer features specialised for fields such as biology chemistry or quality assurance/quality control’. A balance may need to be struck between making an ELN usable across multiple disciplines, whilst still providing enough domain specific knowledge.

Whilst there are many attractive affordances of storing your notes electronically, it is of concern to researchers whether their data is kept truly private or not, once it has been put into these services. Different service providers differ in their privacy policies. For example, Google Docs states that not only do the users maintain intellectual property of any content they create, content will not be shared with any third parties, and the user can take their data with them if they choose to leave Google Docs [20]. However, other services such as Microsoft Office 365, may give contracted third parties access to their customer data (which includes both personal data such as names and email addresses, but also data uploaded into their systems such as images and documents) to perform certain services [29]. An example of a privacy policy controversy is Evernote, where the default was for their employees to be able to read users content to ascertain the accuracy of their machine learning algorithms [44]. It is important for researchers to be aware of privacy policies, and to ensure that research data is secure and can’t be read by third parties when using any software.

### Electronic lab notebooks

**Fig. 1:**
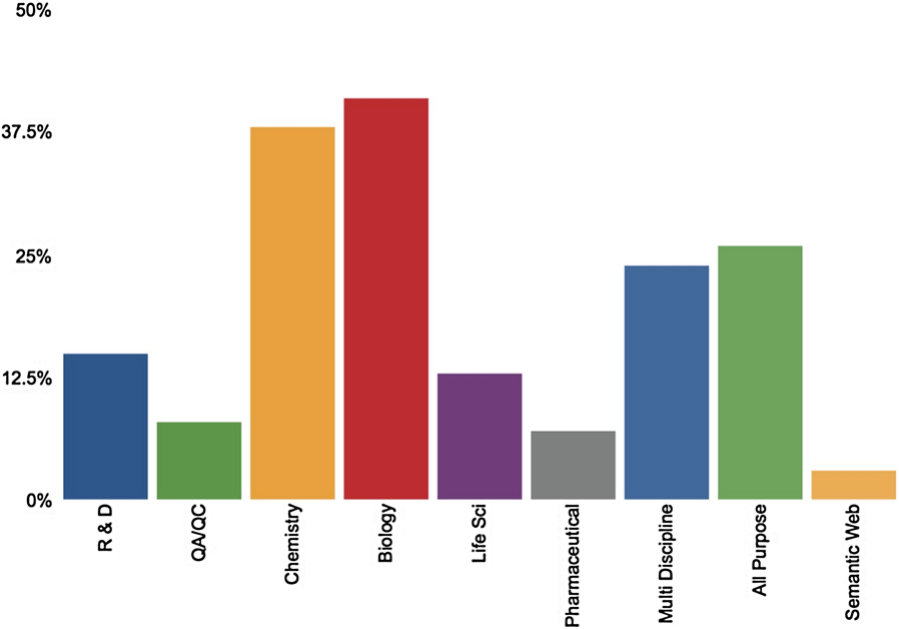
A chart illustrating the different domains represented by the active ELNs in the market

**Fig. 2:**
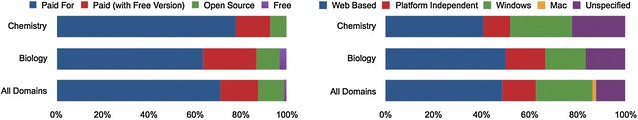
A chart illustrating the licensing and platform information across the active ELNs in the market

Southampton University’s ELN Market study identified 103 ELNs [1, 27, 36, 42], 72 active and 30 no longer active; either due to discontinuation or purchasing by larger companies. The Active ELNs were further investigated to see which domains they supported, and their platform and licensing availability (Figs. 1, 2).

The different licensing categories and associated considerations are as follows:

- Paid for—This is a proprietary piece of software that can be purchased, which may use proprietary data formats.
- Paid (with free version)—This is a proprietary piece of software that can be purchased, but which also has a version of this software which can be used for free; either as a trial for a fixed period of time, or a version that has reduced functionality.
- Open source—This is a product where the code behind the actual software has been made openly available so that anyone can redistribute it and edit it as long as they conform to the licensing conditions. Open Source products are often free, but not always, and could use either standard or proprietary data formats.
- Free—This is a product which is free to use.

These findings illustrate an array of ELNs ranging from supporting specific disciplines (such as eNovalys [15] which is aimed at chemists), to providing all-purpose solutions (such as Kinematik’s eNovator ELN [25] which aims to provide a multi purpose ELN that can be used in many different areas). However, a common factor is that most of these ELNs require payment. Additionally slightly over 60% of them are web based/platform independent, with the rest only available on certain operating systems or without a disclosure of their platform compatibility.

There appears to be a proclivity towards ELNs that make use of pre-existing software. NuGenesis allows users to drag and drop Excel and Word files into their ELN, eLabJournal provides Excel inside it’s ELN, and LIMOSPHY uses Microsoft Word templates. This illustrates an increasing awareness that scientists do use notebooking software, even if they don’t specifically use ELNs. Additionally it suggests that there is a place for ELNs during the final write up process, as well as during the physical experimental process. These ideas will be explored further in “Proposal” section. In addition to the market investigations, current ELN usage was also researched. BioSistemika investigated ELN Usage, and the DaM survey looked at attitudes towards ELNs.

## ELN usage and barriers to adoption

**Fig. 3:**
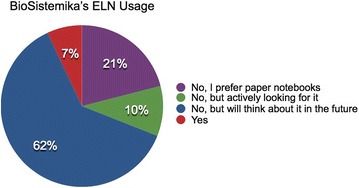
The results of the BioSistemika Webinars: Are you using electronic laboratory notebooks (ELNs) in your Daily Lab Routine?

Despite the saturated ELN Market, results from the BioSistemika and DaM surveys indicated that whilst a large percentage of academic users are considering or interested in using ELNs (as shown in Fig. 3; Table 2), they are lacking in uptake in academia [19, 35]. Many scientists extensively use computers, yet continue to use paper notebooks throughout their experiments; highlighting that computer illiteracy or an aversion to technology cannot fully explain resistance to ELNs [26, 28, 41].

**Table 2:** Attitudes towards ELNs from the respondents of the Dial-a-Molecule’s ‘Potential use of ELNs in Academia’ Survey

ELN sttitudes	%
Awareness of ELNs	98
Using an ELN in their research group	11
Strong interest in implementing one or finding out more about them	76

The University of Southampton’s Lab Practice Study asked users about their ELN usage and experiences. Some participants had used ELNs such as LocalWiki, LabTrove, Blog3, BioBook, Enovalys and an industrial one on a short term basis. The industrial ELN was unfavourably described, whilst the other ELNS were only deemed useful for certain purposes. One participant found Enovalys very useful for inorganic work, but lacking the required functionality for their transport runs. Equally, participants who tried LabTrove and Blog3 found some of the elements useful in certain situations, but all defaulted back to Word documents. One participant suggested that this was the case because it did not contribute in a systematic way to their work.

**Fig. 4:**
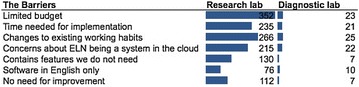
The barriers of using an ELN from both a research lab and a diagnostic lab

There are therefore challenges and barriers to adoption of ELNs. Figure 4 and Table 3 illustrate the key barriers that our studies identified, and these are described in more depth in the following sections.

**Table 3:** Categorised barriers of ELN adoption from the Dial a Molecule iLabber Pilot Project: Potential Uses of ELNs in Academia Survey

Category	Barriers	Percentage of 169 (%)
Cost	Up front costs and licensing fees	74
	Additional infrastructure costs (e.g. computers)	27
	Future development and costs of applications	90
	On-going costs of the system	93
ELN attitude	Only makes sense if the whole department adopts it	20
	Belief that students/post docs would resist adoption	11
Ease of Use	ELN was too difficult to use	22
	Does not capture the right information for me	7
	Difficult to capture some kinds of information in an ELN	80
ELN access	You’d need to enter data in both the lab and write-up area	74
	No easy access to appropriate hardware in the lab	12.5
Data compatibility	Data will be tied into a commercial package	84
Other	Other	11

### Cost

As shown in Table 3, a large percentage of survey respondents indicated that cost was a significant barrier to ELN adoption [4, 19, 35]. This includes financial outlay, staff hours, troubleshooting, and the fact that long-term use is likely to require on-going maintenance and support. There are also concerns about the required database administration and support, with suggestions that having professional IT staff to help with setup and maintenance would be pivotal.

**Fig. 5:**
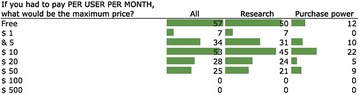
The maximum costs that the respondents of the BioSistemika’s ELN survey would be willing to pay for an ELN per month, from the perspective of those in Research, and with Purchasing Power

One respondent experienced sharp price-increases in database maintenance and upgrade costs after an initial discount. Other concerns are service providers not competing to keep costs down, and the potential cost of storage space; indicating disincentives if the University charges groups for storage space. Figure 4 illustrates a willingness to pay up to $50 a month for an ELN, but not $100; suggesting that ELNs reach a point where they are considered ‘too expensive’ (Fig. 5).

Other comments queried whether funding was available for ELNs in universities; suggesting that web-based systems could significantly cut costs, as they require less hardware.

As evidenced by Fig. 2, most of the ELNs available in the market are proprietary pieces of software that require purchasing, there are also some free and open source offerings available. The free options would clearly have the advantage of being cheaper to run and test but may have disadvantages depending on the nature of the software. The paid for and free software model (one of the categories in Fig. 2) will have enterprise users to generate its revenue, and are able to offer reduced free versions to other users to generate recognition; providing the benefits and stability of proprietary software with a potential lack of cost. However, the fear is that other standalone free offerings are more likely to disappear, potentially alongside the research data. Some of the inactive free ELNs that were identified as part of the University of Southampton’s ELN Study were listed on [1, 24] with websites that had seemingly vanished with no new obvious location. Open Source software is often free (although not always) and has a significant advantage over proprietary software with respect to their potential longevity. Both Open Source and Proprietary projects will always be at risk of ceasing to continue, either due to lack of funds or the original developers leaving the project. However, given the licensing of Open Source projects which makes it possible to view and change the source code, other developers are able to access, update and support the software.

### Ease of use

Another challenge is the perceived ‘ease of use’ of paper notebooks compared to electronic systems. Paper notebooks are considered easier to use, input data to, read, transport, inexpensive, readily available, ‘turn on’ instantly, have infinite battery life, are socially acceptable during meetings, and require no training and minimal IT support [4, 8, 11, 19, 28, 50]. Whereas, ELN software for taking notes is considered more difficult to use, timely and less flexible; leading to anxieties about ELNs stability, accessibility and availability [14].

Our interactions with researchers and ELN users demonstrate that ease of use is vital to adoption. In the DaM survey, 99% of respondents indicated that ease of use would influence their ELN choice, with almost 80% rating it as very important. One comment reflected the desire for a flexible generic solution, rather than an ELN designed for a specific research area, due to anxieties that their research “doesn’t fit neatly into one category”.

### Attitudes to ELNs

Adopting an ELN only makes sense if the whole department adopts it, which allows for sharing costs and training; repositories, consistency and use of standards could also be relevant [10, 42]. Several comments reflected their assumption of students and postgrads rejecting ELNs through “resistance to change in some groups”, noting that some students didn’t like their experiences of ELNs, and might consider using them as an “additional burden”.

### Access to ELNs

In the Uses of ELNs in Academia survey, 74% expressed concerns about needing to enter data in both the lab and write-up area, due to a lack of suitable hardware or software capabilities to facilitate ELN usage inside and outside the lab. This can lead to copying and pasting printouts into paper notebooks and manually transcribing data between notebooks and computers; which can result in data loss, transcription errors and records stored haphazardly [9, 32]. Popular suggestions were to use mobile computers or tablets for portability in and out of the lab, and that web-based ELNs could improve accessibility.

Lack of appropriate hardware access in the lab lead to 12.5% of participants in the Post Pilot Survey ceasing to use the trialled ELN, and resulted in several needing to perform tasks manually. Other anxieties frequently raised, included risk of damage or contamination, security, ‘hassle’ of carrying laptops around, shortage of computers for sharing, lack of bench space for computers, ELN not supported on chosen mobile platform, and lack of wifi access. Primary workarounds for these issues appeared to be printing out experiments, writing up experiments retrospectively, or using paper notebooks alongside the ELN.

### Software and system integration and compatibility

Researchers use different operating systems, but both the ELN Market study and comments from the DaM survey revealed a lack of availability of ELNs for Macs. It was suggested that iPads could work as a shared notebook due to their ease of transport, although software would need to be compliant with iOS and other mobile platforms, or web based. A perceived barrier was linked to integrating ELNs with existing infrastructures. The DaM Surveys had similar concerns that users might be expected to purchase new ELN software at each operating system upgrade; which could contribute to system costs, support costs, and additional training requirements.

Electronic pen data entry, integration with digital repositories for archiving purposes and bibliographic management have also been mentioned with regards to integration with existing tools. The DaM Pilot Program elicited a need for software compatibility, database integration, electronic data, and other ‘common software’ (e.g. Word and Excel), and options to purchase add-ons for increased functionality. Users found problems using the ELN on a 64-bit operating system and on macs, or with Chemdraw, office attachments, uploading photographs, and “…it was too cumbersome to import files from our current systems…”. ELN data input seems to have been a recurring issue, alongside failings in basic expectations about data management that heightened existing anxieties.

### Data compatibility and portability

In the DaM Pilot survey just under 70% expressed concern about the ELN capturing information easily, with 81% considering automatic experiment data capture important. Comments indicated that capturing a range of data is important, but raised concerns about the difficulties of instrument integration, partly due to a lack of standards between different manufacturers. Many comments expressed frustrations about not being able to link to specific experimental data such as spectroscopic results.

Several comments indicated worries about the ability to extract and move data between different ELNS and machines; these concerns relate to price hikes with a provider, longevity of commercial packages, and changing institution. Other comments addressed issues of proprietary formats including previous bad experiences of “being tied into data formats” or being left with only a PDF of their data; although the desired transferral formats differed between respondents. Some comments embraced the importance of open data and not being tied to a particular commercial package, suggesting an open source ELN to resolve the problem. This suggests that researchers perceive open source offerings to be more likely to use standard data formats rather than proprietary formats. Concerns were also expressed regarding accessing databases and notebooks across different machines, suggesting that users expect their information to be stored locally or in a centralised system, and are concerned about data security.

## What do users do?

What users say they do doesn’t always match their actions; therefore after establishing the main adoption barriers, we investigated how the researchers actually worked. Four focus groups were run with 24 postgraduate chemists, physicists and biologists to discuss their current practices. Additionally, four different chemistry labs were observed to see how scientists operated there.

### Results

We found that different researchers vary their working patterns and note-taking, and have contrasting needs when it comes to sharing records with others. Therefore a ‘one size fits all’ approach to tool design wouldn’t be effective. Tools need to provide considerable flexibility and customisation to accommodate different needs. The high level results of these activities across the different disciplines are presented in Table 4, and will be further discussed later on in this section.

**Table 4:** How the physicists, chemists and biologists who took part in the University of Southampton Focus Groups perform different work tasks with respect to whether they use paper or electronic systems

Category	Tasks	Biologists	Chemists	Physicists
Recording notes	Experiment notes	Paper—Lab Book	Paper—Lab Book Electronic—Data	Paper—Lab Book Electronic—Data
	Thinking about work notes	Paper—Lab Book	Paper—Lab Book Electronic—Google Tasks	Paper—Lab Book Electronic—Google Keep
	Literature notes	Paper—Print papers/handwritten notes Electronic—reference manager	Paper—Print papers/handwritten notes Electronic—reference manager	Paper—Print papers/handwritten notes Electronic—reference manager
Organising notes		Paper—Lab Book by date/contents page	Paper—Lab Book by date/contents page Electronic—By codes (linking to Lab Book) and by sample/experiment	Paper—Lab Book by date/contents page Electronic—by codes (linking to Lab Book) and by category/experiment
Searching		Paper—flip back and search by date	Paper—flip back and search by date Electronic—sort by date/code, or keyword search	Paper—flip back and search by date Electronic—sort by date
Linking data		Paper and Electronic notes linked by date	Paper and Electronic notes linked by codes	Paper and Electronic notes linked by date
Writing reports		Electronic—Word/Powerpoint	Electronic—Word/LaTeX	Electronic—word/LaTeX
Performing calculations and scientific functionality		Paper—solve Electronic—check (Excel/GraphPad)	Paper—solve Electronic—Check (Wolfram Alpha)	Paper—solve Electronic—Check (Excel/XMGrace/Spartan/PyPlots/R/CSV)
Use of Technology in the Lab (accessibility)		Electronic—Phone pictures/recordings	Electronic—Phone/camera pictures, Emails, Blogs, USB	Electronic—Phone pictures/calendar, Emails
Archiving and backup		Paper—Mostly no backup (some photocopies) Electronic—Uni computers/shared drives/the cloud/hard drives	Paper—Mostly no backup (some use carbon pages) Electronic—Uni computers/shared drives/the cloud/hard drive	Paper—No backup Electronic—Uni computers/shared drives/the cloud/hard drives
Intellectual property		Electronic—Secure data kept on hard drive in locked draw	Electronic—No cloud software for industry sponsored students	Electronic—No cloud software for industry sponsored students
Collaboration		Paper—Lab Book	Paper—Lab Book Electronic—shared drive/group folders	Paper—Lab Book Electronic—shared drive/group folders

### Discussion

The biologists and physicists from these focus groups were mostly uniform in their methods, whereas the chemists were more diverse, highlighting differences in their approach even within a single discipline.

Computational chemists used some software, with sporadic use of lab books and scraps of paper, whereas the ‘wet’ chemists had stringently organised lab books for different tasks. One chemist used blogs and Word documents alongside their paper notebooks, and the crystallographers relied heavily on their paper sample books. The inorganic and organic chemists used paper lab notebooks during experiments, and only used lab computers to access the instruments they were linked to. Note-taking differed depending on the situation. For experiments, the lab book was typically used to record observations and initial values. The chemists recorded different types of data including energy values, simulations, temperature, masses, observations, schemas, and protocols. These findings have similarities to Reimer and Douglas’s [34] work, illustrating how information recorded remained much the same; but also demonstrating that different chemists possess contrasting needs for recording their notes. Constructing one’s own ‘templates’ or other mechanisms for standardising data capture appears to be common in academic environments [40]; therefore providing capabilities to facilitate this is likely to be popular [2, 40]. Allowing users to edit their own templates poses challenges. This is illustrated by a comment from one of the lab observation participants, who resented being asked to use a template rather than expressing themselves in their own style.

Chemists also differed in how they linked their paper and electronic notes. The physicists and biologists linked them by date, whereas the chemists inconsistently used a variety of codes; reflecting the personal nature of note organisation [40]. Despite their differing lab work, there was a common theme of using instruments (e.g. X-ray machines or diffractometers) to read data, and linking statements in their lab book to reference electronic data location and any data values that required inputting to other software. In some situations it may be necessary to capture some information on paper, and ELNs therefore need to facilitate the inclusion of such information with the research record.

Different disciplines had varying restrictions on what equipment could be taken into the lab. The biologists didn’t have specific restrictions, although one biochemist mentioned that there were concerns about bringing in outside equipment in case of contamination. The physicists couldn’t bring equipment into their cleanroom to avoid contaminating the environment; contrastingly, the chemists wouldn’t take technology into the lab in order to avoid damaging it with chemicals. Computers in the lab weren’t often online, and most were connected to specific instruments. When asked, participants indicated a reluctance to use instrument dedicated computers for any other purpose, such as making notes, accessing documents remotely, or using cloud software as they didn’t have network access. One chemist stated that “once you’ve started doing something one way, you don’t want to change it”.

### Scenarios

**Fig. 6:**
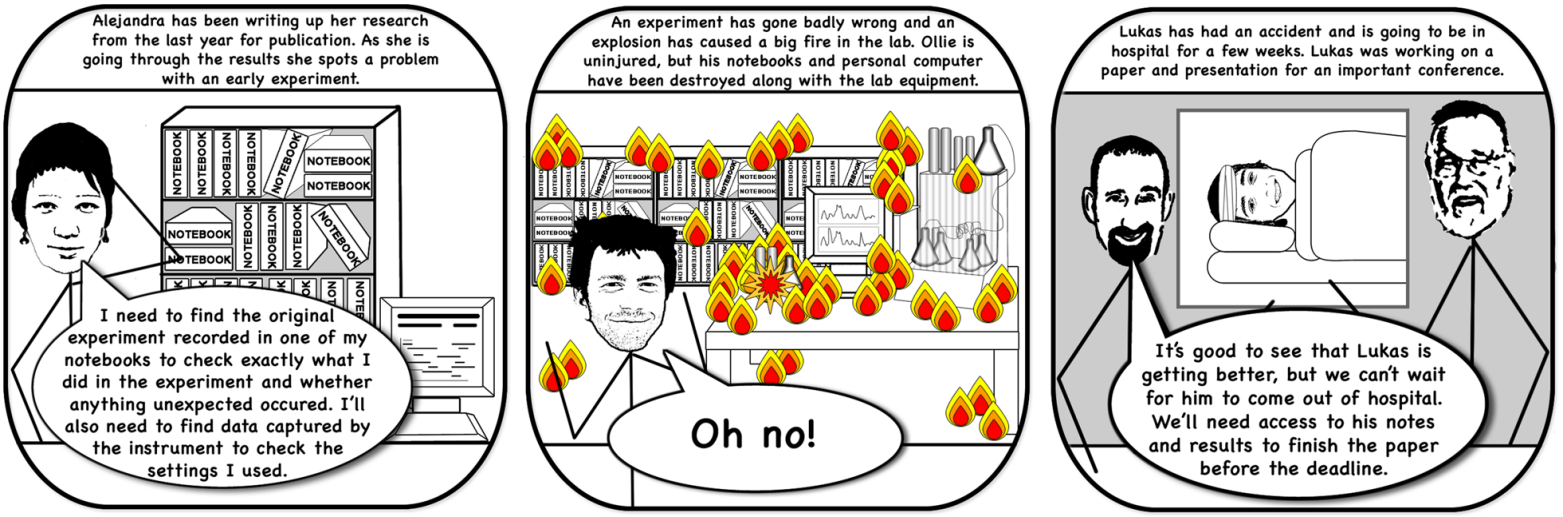
Cartoon depicting three different scenarios, Scenario 1: Trying to search for some work/data 6 months later, Scenario 2: What would happen if your lab was set on fire and you lost everything in there, Scenario 3: If you were indisposed for a while how would your supervisor/research group access your work

To investigate the participant’s current searching and backup procedures, they were presented with three scenarios to discuss (illustrated in Fig. 6):

- Imagine you’re trying to locate some work from 6 months ago, how would you locate you notes and associated data?
- Imagine there’s a fire in your lab and all of your paper notebooks are destroyed, how much work would you lose and how could you go about recovering it?
- If you fell under a bus tomorrow, and were temporarily indisposed, how would your supervisor/industry sponsors/colleagues access your work?

For Scenario 1, participants revealed that they organised their lab books chronologically, and the most common method of locating previous work was to go back through their lab book by date to locate work from a particular time period. Similarly to locate previous work on a computer participants said that they would search by date to find the appropriate data files, or would search by name if that proved unsuccessful.

Scenario 2 provoked different reactions. Some participants were unconcerned at the prospect of losing their lab books, and thought reproducing what was needed wouldn’t take too long, as a lot of the information was only ‘useful in the moment’, or a list of things that didn’t work. Whereas other participants elicited responsed such as ‘I’d be ruined’, ‘a nightmare’, ‘might as well stop my Ph.D. now’. Particularly with reference to the idea of their labs catching fire, several participants seemed more concerned at the idea of losing their lab samples or compounds; suggesting that perhaps their lab books would not be the biggest loss in a fire.

Scenario 3 revealed that generally participants don’t have measures in place to enable their supervisors to access their work if something happened to them. One of the biologists had a particularly strict supervisor who required their students to photocopy all of their lab books and work, but that was a rare exception. It did however transpire that the participants believed that other group members would probably be able to access their work and give it to their supervisors, but didn’t believe that they would be able to follow their lab books or the structures they’d put in place to link together their paper and electronic notes.

This continues the earlier theme about participants showing less concern towards backing up their paper based work. They are obviously aware that these scenarios could occur, but clearly don’t perceive them as likely or serious enough to merit much pre-emptive preparation, apart from circumstances where their supervisors have put procedures in place. Capturing notes and data electronically has clear backup and archiving benefits. Not only can electronic information be automatically backed up and securely stored, but the information can become accessible across multiple locations. Outdated information can be archived so that it can be retrieved later if needed, or to be shared with other researchers through deposition or publication.

## What do users want?

Having discussed with the users what they actually do, this section will look at what features the users say they want, with information taken from all studies B, D, E and F.

These have been grouped according to the different categories in “What do users do” section in addition to a new category of project activities that came out of this research. These features have also been linked to the associated priorities of the iLabber pilot project for those who found these features very or quite important; and it’s been noted which barriers these features aim to address.

**Fig. 7:**
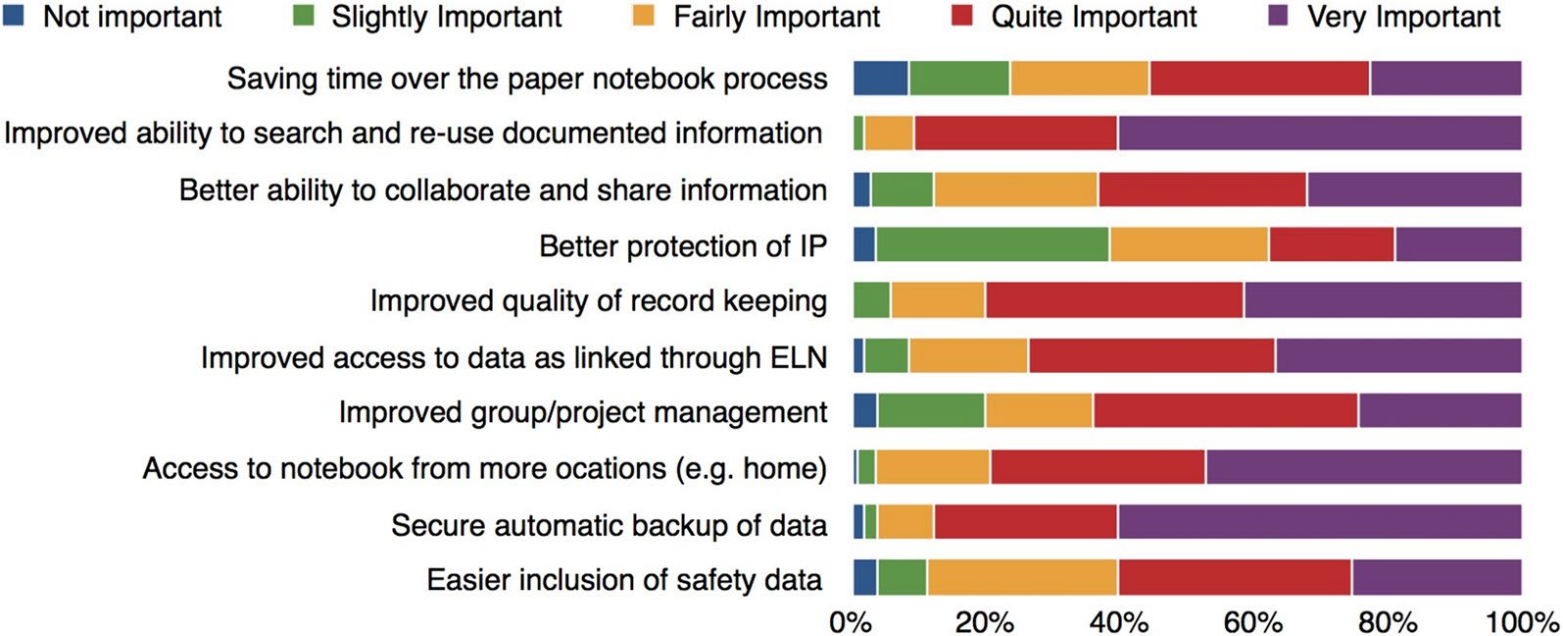
The main priorities of different ELN features from the respondents of the iLabber Piilot Project, ranging from whether respondents saw them as not important to very important

The full breakdown of priorities from the iLabber Pilot Project are shown in Fig. 7.

## Proposal

Based on the needs elicited from our user studies, we formulated a proposal of how to construct an ELN environment that would fit with these requirements. The majority of ELNs have been created from scratch including the underlying ‘notebook’ part [31, 32, 42]; an alternative would be to build on top of a generic Electronic Notebook (which are more popular than ELNs) with domain specific features. Many of these Electronic Notebooks already have collaborative cloud based features, and could be further expanded with domain knowledge and Semantic Web technologies. Additionally, based on our market research, despite the amount of available ELNs there are a minority that are available as free/open source platform independent entities that scientists can use on any device, suggesting a gap that could be filled with this type of ELN.

### Visions

As part of this vision BioSistemika used their ELN survey to create their own ELN, sciNote [38]. Taking the path towards interoperability, sciNote has been designed in a modular way and released under the Open Source licence (Mozilla Public Licence). Based on the user needs they are developing new add-ons and at the same time they are encouraging the community to develop their own add-ons, similar to the packages concept of R-statistical. In this way every lab will be able to design their own ELN to fit their needs, which will help them manage their project, share research, gather the metadata directly from instruments and connect with existing software and databases.

Southampton University has looked at the features the users want (shown in Table 5) and formulated how these could be achieved using an Electronic Notebook Platform as a base. There is a large overlap of features between Electronic Notebooks, ELNs and SLNs and the main features required by an ELN already exist in generic Electronic Notebooking software [34]. Furthermore, using a cloud based Electronic Notebook platform would combat some of the accessibility issues and facilitate the collaboration requirements of the users, and incorporating Semantic Web technologies would provide an improved (semantic) search (the top priority listed in Fig. 7) and allow for metadata/tagging (as requested).

**Table 5:** These are the desired features elicited from the different user studies, linked to the priorities and barriers they relate to from the Dial-a-Molecule surveys

Category	Desired Features	Priorities (DaM)/Addressing Barriers
Recording notes	Simple to install Personalisable Post-it notes TODO lists Create default values Easy to write in as a paper notebook Facilitate different experiments Range of experiment templates	55.6%—Saving time over the paper notebook process is important Barrier: Ease of use (3.2)
Organising notes	Indexable/highlightable Contents table/overview screen/timeline Spellchecker Tag/classify notes and experiments Store metadata Use of standard vocabularies (ontologies/measurement techniques)	80.2%—Improved quality of record keeping is important
Searching	Keyword/filtered search Data traceability Advanced searches by chemical structure Include reactions schemes in search results Voice searches Sortable results	90.6%—Improved ability to search and re-use documented information is important
Linking data	Upload/link files, images and data files to notes Link between different notebooks Link to reference managers Dropbox-esque features (automatic data update) Automatically link to external chemistry resources	73.6%—Improve access to data as linked data through ELN is important Barriers: Data Compatibility and Portability (3.6)
Writing reports	‘Generate Report’ button to generate a publication ready report Integrate and store different types of documents (Excel, Word, PDF, Pictures, Handwritten notes) Copy sketches into notebook Paper notebooks integration Digital pen integration Migration tools Export functionality	Barrier: software and system integration and compatibility (3.5)
Performing calculations and scientific functionality	Perform calculations, formulas and equations as easily as paper Create sketches and diagrams Recognise a chemical when entered Risk Assessment Templates/view electronically Flags for dangerous chemicals Index of COSHH materials Global database of chemical values Notifications for approvals Sign off entries to make them non editable	60.4%—Easy inclusion of safety data is important
Use of Technology in the Lab (accessibility)	Web Based/Platform Independent Tablet/Smartphone Compliant Text recognition, drawing and photo capabilities Usable in the lab like a paper notebook Voice capture Built in language for extensibility	79.3%—Access to notebook from more locations is important Barrier: Access (3.4)
Archiving and backup	Secure storage, backup and archives Downloads/printing	87.8%—Secure automatic backup of data is important
Intellectual property	Secure access Different access levels for users	37.8%—Better protection of IP is important
Collaboration	Shared files/notebooks Standard list of instruments and reagents Link related people and notebooks Coordination for Open Source and Access Sign up and ‘get involved’ pages Configurable stand-alone to act as portals for projects and landing pages for collaborators Enable users to find out who is working on similar molecules of reactions (requires inbuilt understanding of molecules)	63.2%—Better ability to collaborate and share information is important
Project activities	Recent activity feed with notifications Page statistics Bulletin boards Moderate comments	64.1%—Improved group/project management is important

### A cloud based ELN

There will always be concerns about IP with regards to using Cloud based services. The University of Southampton’s Lab Practice study elicited that users with industry sponsors were less likely to use Cloud software. It is thought that once data is ‘in the cloud’ users are no longer in control [13], and that like with any electronic service there is the potential for data breaches [45] however many precautions are taken. However this concern certainly isn’t restricted to electronic data. Some of the biologists from the University of Southampton’s Lab Practice Study said that they didn’t consider their work to be safe at conferences as people may take photographs of their posters and steal their ideas, and some of the chemists were aware that previous members of their research group had been ‘scooped’ which resulted in tightened security measures across the group. Despite this, cloud computing is advantageous in that it can provide large volumes of storage and computing power that are accessible from any location [13, 45], and it’s worth noting that only 18.9% of respondents in the iLabber Pilot Project Survey thought that ‘Better protection of IP’ was ‘Very important’, ranking significantly below Improved search and secure automatic backup of data, both of which lend themselves greatly to our proposed methods.

### Proposed features/design

**Fig. 8:**
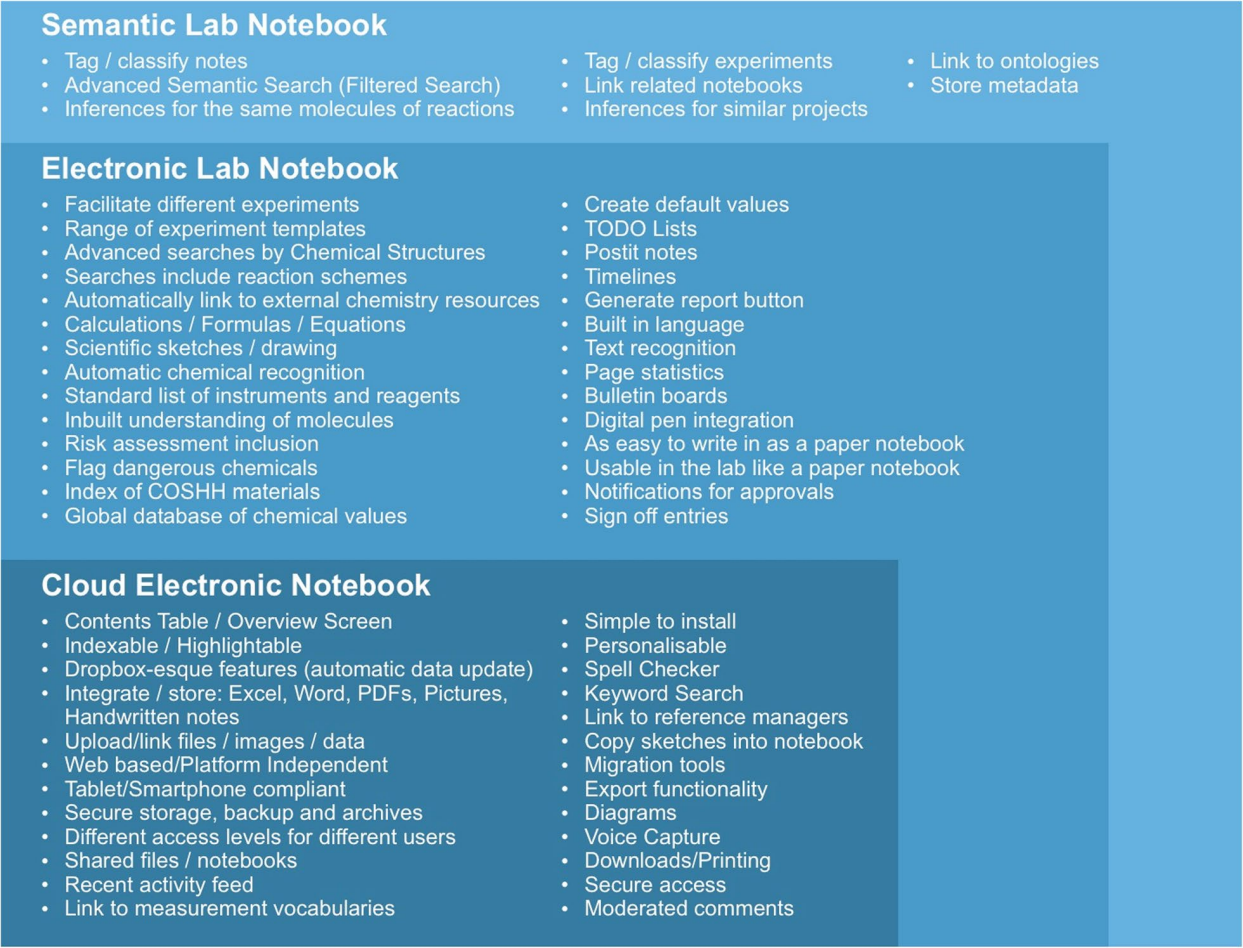
The desired features that have been elicited from the different user studies. Categorised by whether they are features already included in a cloud based notebook, and then whether they fall into the category of an ELN domain specific feature or a semantic feature

When investigating the features our users want, we realised that approximately 40% of these features are already implemented within cloud based electronic notebooking software, and the rest of the features are either domain specific or could be achieved using semantic web technologies. Figure 8 shows these desired features elicited from our user studies detailed in Table 5, which are supported by previous ELN research work [4, 18, 22, 34, 37, 42, 46].

We believe that this approach and the subsequent ELN environment that will be developed can mitigate the current barriers and concerns, these are detailed in Table 6.

**Table 6:** How the proposed system could mitigate the barriers elicited from the Dial a Molecule iLabber Pilot Project: potential uses of ELNs in Academia Survey

Barrier	Mitigation
Cost (3.1)	ELN would be free
Ease of use (3.2)	Using a pre-existing Electronic Notebook would mean users are already be familiar with the system and rather than building the notebooking side from scratch it would use a tried and tested product
Attitudes to ELNs (3.3)	Adding a domain/semantic layer to software scientists already use might improve attitudes towards this type of ELN
Access to ELNs (3.4)	A cloud based ELN can be accessed anywhere with an internet connection on any desktop or mobile device (including phones and tablets)
Software and system integration and compatibility (3.5)	Cloud software is platform independent
Data compatibility and portability (3.6)	Using cloud software to store data means it can be accessed across multiple devices. The cloud notebook would allow the user to export their research data in a variety of common data formats.

**Fig. 9:**
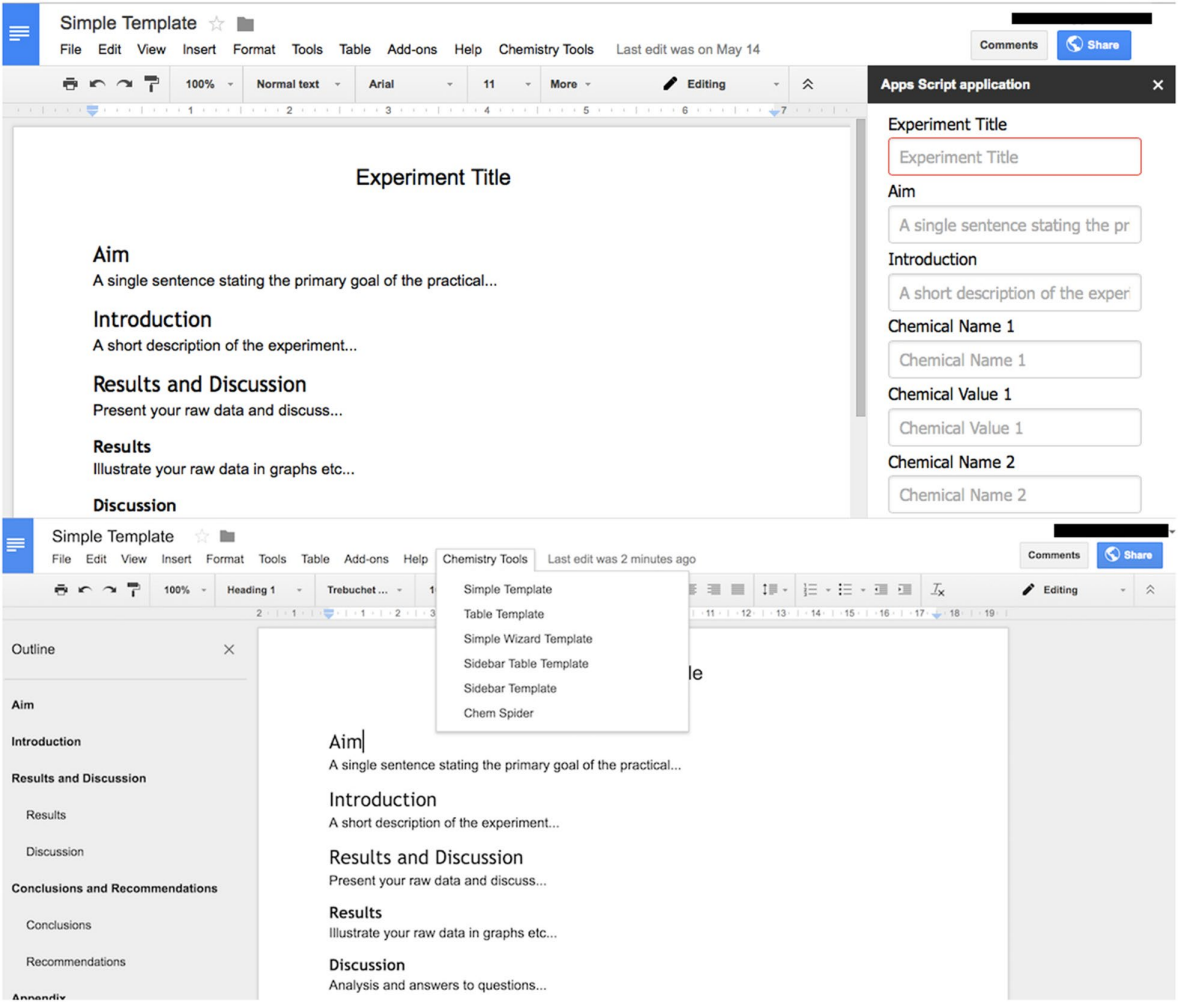
An example of adding domain specific features to a pre-existing cloud notebook tool

Therefore we propose that building a semantic ELN on top of an existing cloud infrastructure or platform would allow us to make use of these pre-existing features, provide a solid notebook base aligned with software scientists already use, and would also help combat the current adoption barriers. The ability to adapt documents and control input provided by a platform such as Google Docs enables much of the functionality needed for an ELN (e.g. in Fig. 9).

## Conclusions and future work

Our user studies have made one thing very clear, we cannot currently hope to fully replace the paper lab notebook. Until we have the technology where a screen can be written on as accurately and easily as paper; and labs have cheap, durable and easily replaceable tech to use instead of paper, it will always prevail for some tasks. We also need to stop thinking of ELNs as direct replacements for paper lab notebooks that are only useful during experiments in the lab, and consider them in the wider context of the whole experiment process. Therefore we need to build a system that works with paper, and formulate a new digital practice for scientists to use in their current lab environment.

Despite some scientists preferring paper notebooks, they still frequently use technology in their work. Many store data electronically, and use note-taking software such as Word and Evernote to write up their notes, Excel to handle their figures and graphs, and some use speciality software for specific tasks. The cloud is also widely used to backup work and make it available across different locations. Therefore we need to start considering ELNs that can work in this context, and to work out how we can re-use existing successful software to create a better ELN platform.

We propose that we need an ELN environment that can serve as an interface between paper lab notebooks and the electronic documents that scientists create; that is interoperable and utilises Semantic web and cloud technologies. It would fulfil all of the software needs described in “What do users want” section and provide a centralised location for the scientists to store their notes. Whilst the ideal long term goal is that adoption of an ELN alongside extensive laboratory automation removes any need for paper, realistically current technology is such that it is desirable that ELN solutions work alongside paper for the foreseeable future. We believe that ELNS will significantly improve reproducibility of scientific experiments, contribute to the data traceability and data annotation and enable scientists to collaborate and share results in an intuitive manner. The wider adoption of ELNs will facilitate interoperability which will ultimately change the ways scientists perform experiments and manage their data. There’s a great potential for future work in these areas, as an ELN that follows our vision has yet to be created, and as hardware and technology as a whole advances, we will be able to support even more of the experimental process digitally.

## Additional file

**Additional file 1.** A file describing how to access the focus groups and lab observations transcripts from Study D—University of Southampton Lab Practice Study (Focus Groups & Lab Observations).

## Abbreviations

DaM: Dial-a-Molecule
ELN: electronic lab notebook
ELNs: electronic lab notebooks
IP: intellectual property
RDF: resource description framework
SLNs: semantic lab notebooks

## References

[1] Atrium Research. Electronic Laboratory Notebook. http://www.atriumresearch.com/eln.html. Accessed 7 Jan 2016

[2] Badiola KA, Bird C, Brocklesby WS, Casson J, Chapman RT, Coles SJ, Cronshaw JR, Fisher A, Frey JG, Gloria D, Grossel MC (2015). Experiences with a Researcher-Centric ELN. Chem Sci.

[3] BioSistemika. Software development partner for life sciences. http://biosistemika.com/. Accessed 7 Jan 2017

[4] Bird CL, Willoughby C, Frey JG (2013). Laboratory notebooks in the digital era: the role of ELNs in record keeping for chemistry and other sciences. Chem Soc Rev.

[5] Borkum M, Lagoze C, Frey J, Coles S (2010) A semantic eScience platform for chemistry. In: 2010 IEEE sixth international conference on e-Science (e-Science). IEEE, pp 316–323

[6] Borman S (1994). Electronic laboratory notebooks may revolutionize research record-keeping. Chem Eng News.

[7] Boulton G, Campbell P, Collins B, Elias P, Hall W, Laurie G, O’Neill O, Rawlins M, Thornton J, Vallance P, Walport M (2012). Science as an open enterprise.

[8] Brandl P, Richter C, Haller M (2010) Nicebook: supporting natural note taking. In: Proceedings of the SIGCHI conference on human factors in computing systems. ACM, pp 599–608

[9] Bruce S (2008) A look at the state of electronic lab notebook technology. Sci Comput. http://www.scientificcomputing.com/article/2002/12/look-state-electronic-lab-notebook-Technology. Accessed 7 January 2017

[10] Coles SJ, Frey JG, Bird CL, Whitby RJ, Day AE (2013). First steps towards semantic descriptions of electronic laboratory notebook records. J Cheminform.

[11] Cooke R, Schraefel MC (2004) Signature flip and clip: virtually flipping and dog earing pages in a Digital Lab Book. In: Proceedings of the user interface software (UIST), Fante Fe, USA, Mexico. http://eprints.soton.ac.uk/259251/. Accessed 7 Jan 2017

[12] Coppin P, Hockema SA (2009) Learning from the information workspace of an information professional with dyslexia and ADHD. In: 2009 IEEE Toronto international conference on science and technology for humanity (TIC-STH). IEEE, pp 801–807

[13] di Vimercati SD, Foresti S, Samarati P (2015) Data security issues in cloud scenarios. In: International conference on information systems security. Springer, pp 3–10

[14] Drake DJ (2007). ELN implementation challenges. Drug Discov Today.

[15] Enovalys. http://www.enovalys.com/book. Accessed 16 May 2017

[16] Evernote. https://evernote.com/. Accessed 7 Jan 2016

[17] Fakas GJ, Nguyen AV, Gillet D (2005). The electronic laboratory journal: a collaborative and cooperative learning environment for web-based experimentation. Comput Support Cooper Work (CSCW).

[18] Frey JG, De Roure D, Mills H, Fu H, Peppe S, Hughes G, Smith G, Payne TR (2017) Context slicing the chemical Aether. http://eprints.soton.ac.uk/258790/. Accessed 7 Jan 2017

[19] Goddard NH, Macneil R, Ritchie J (2009). eCAT: online electronic lab notebook for scientific research. Autom Experiment.

[20] Google. Google Privacy Terms. https://www.google.com/intl/en/policies/terms/. Accessed 16 May 2017

[21] Hice RC (2009) Roadmap to a clear definition of ELN. Sci Comput. http://www.scientificcomputing.com/blog/2009/05/roadmap-clear-definition-eln. Accessed 7 Jan 2017

[22] Hughes GV, Mills HR, Smith G, Payne TR, Frey J (2004) Breaking the book: translating the chemistry lab book into a pervasive computing lab environment. In: Proceedings of the SIGCHI conference on human factors in computing systems. ACM, pp 25–32

[23] Hughes G, Mills H, De Roure D, Frey JG, Moreau L, Smith G, Zaluska E (2004). The semantic smart laboratory: a system for supporting the chemical eScientist. Org Biomol Chem.

[24] Kaiser J (2017) Rigorous replication effort succeeds for just two of five cancer papers. http://www.sciencemag.org/news/2017/01/rigorous-replication-effort-succeeds-just-two-five-cancer-papers?utm_source=newsfromscience&utm_medium=facebook-text&utm_campaign=cancerrep-10608. Accessed 3 Feb 2017

[25] Kinematik (2017) http://www.kinematik.com/solutions/industry/electronic-laboratory-notebook. Accessed 16 May 2017

[26] Klokmose CN, Zander PO (2010). Rethinking laboratory notebooks. Proc DISC.

[27] LIMSwiki. ELN Vendor. http://www.limswiki.org/index.php?title=ELNvendor. Accessed 7 Jan 2016

[28] Mackay WE, Pothier G, Letondal C, Bøegh K, Sørensen HE (2002) The missing link: augmenting Biology Laboratory Notebooks. In: Proceedings of the 15th annual ACM symposium on user interface software and technology. ACM, pp 41–50. doi:10.1145/571985.571992. Accessed 7 Jan 2017

[29] Microsoft. Office 365 Privacy Statement. https://www.microsoft.com/online/legal/v2/?docid=43. Accessed 16 May 2017

[30] Microsoft. Onenote. https://www.onenote.com/. Accessed 7 Jan 2016

[31] Mohd Zaki Z, Dew PM, Lau L, Rickard AR, Young JC, Farooq T, Pilling MJ, Martin CJ (2013). Architecture design of a user-orientated electronic laboratory notebook: a case study within an atmospheric chemistry community. Future Gen Comput Syst.

[32] Myers JD, Mendoza ES, Hoopes B (2001) A collaborative electronic laboratory notebook. In: IMSA, pp 334–338. http://pdf.aminer.org/000/877/371/computational_experiments_using_distributed_tools_in_a_web_based_electronic.pdf. Accessed 7 Jan 2017

[33] Oleksik G, Milic-Frayling N, Jones R (2014) Study of electronic lab notebook design and practices that emerged in a collaborative scientific environment. In: Proceedings of the 17th ACM conference on computer supported cooperative work and social computing, pp 120–133. ACM. doi:10.1145/2531602.2531709. Accessed 7 Jan 2017

[34] Reimer YJ, Douglas SA (2004). Ethnography, scenario-based observational usability study, and other reviews inform the design of a web-based E-notebook. Int J Hum–Comput Interact.

[35] Rudolphi F, Goossen LJ (2011). Electronic Laboratory Notebook: the academic point of view. J Chem Inform Model.

[36] Rubacha M, Rattan AK, Hosselet SC (2011). A review of electronic laboratory notebooks available in the market today. J Assoc Lab Autom.

[37] Schraefel MC, Hughes G, Mills H, Smith G, Frey J (2004) Making tea: iterative design through analogy. In: Proceedings of the 5th conference on designing interactive systems: processes, practices, methods, and techniques, pp 49–58. ACM. doi:10.1145/1013115.1013124. Accessed 7 Jan 2017

[38] sciNote. Free open source electronic lab notebook. http://scinote.net/. Accessed 7th Jan 2017

[39] Shankar K (2004). Recordkeeping in the production of scientific knowledge: an ethnographic study. Arch Sci.

[40] Shankar K (2007). Order from chaos: the poetics and pragmatics of scientific recordkeeping. J Am Soc Inform Sci Technol.

[41] Tabard A, Mackay WE, Eastmond E (2008) From individual to collaborative: the evolution of prism, a hybrid laboratory notebook. In: Proceedings of the 2008 ACM conference on computer supported cooperative work. ACM, pp 569–578. doi:10.1145/1460563.1460653. Accessed 7 Jan 2017

[42] Talbott T, Peterson M, Schwidder J, Myers JD (2005) Adapting the electronic laboratory notebook for the semantic era. In: Proceedings of the 2005 international symposium on collaborative technologies and systems, pp 136–143. IEEE. doi:10.1109/ISCST.2005.1553305. Accessed 7 Jan 2017

[43] Taylor KT (2011). Evolution of electronic laboratory notebooks. Collab Comput Technol Biomed Res.

[44] TechCrunch. Evernote’s new privacy policy allows employees to read your notes. https://techcrunch.com/2016/12/14/evernotes-new-privacy-policy-allows-employees-to-read-your-notes/. Accessed 16 May 2017

[45] Thiebes S, Dehling T, Sunyaev A (2016) One size does not fit all: information security and information privacy for genomic cloud services. ECIS 2016 Proceedings

[46] Voegele C, Bouchereau B, Robinot N, McKay J, Damiecki P, Alteyrac L (2013). A universal open-source electronic laboratory notebook. Bioinformatics.

[47] Walsh E, Cho I (2013). Using Evernote as an electronic lab notebook in a translational science laboratory. J Lab Autom.

[48] Willoughby C, Frey JG. From paper notebooks to digital research management: designing digital tools for laboratory scientists. In Preparation

[49] Wilson DF (2017) Purposive variation in recordkeeping in the academic molecular biology laboratory. Doctoral dissertation, University of Glasgow. http://theses.gla.ac.uk/2482/. Accessed 7 Jan 2017

[50] Yeh R, Liao C, Klemmer S, Guimbretière F, Lee B, Kakaradov B, Stamberger J, Paepcke A (2006) ButterflyNet: a mobile capture and access system for field biology research. In: Proceedings of the SIGCHI conference on human factors in computing systems. ACM, pp 571–580

