# Supplementary material for: Electronic lab notebooks: can they replace paper?
Source: J Cheminform. 2017 May 24;9:31. doi: 10.1186/s13321-017-0221-3 (PMC5443717; doi:10.1186/s13321-017-0221-3)
Supplement: Supplementary file 1 — Additional file 1. A file describing how to access the focus groups and lab observations transcripts from Study D—University of Southampton Lab Practice Study (Focus Groups & Lab Observations). [file 13321_2017_221_MOESM1_ESM.zip › ElectronicSupplementaryData/DatasetD-FocusGroupQuestions.pdf]

ELN Focus Group

1. What method do you use to record your notes?

2. For each of these following different types of work, what pieces of information do you currently record, and how do you record it (e.g notes, mind maps, graphs, pictures, photos, diagrams, tables etc)
    a. Doing an experiment in the lab
    b. Doing an experiment outside of the lab
    c. Looking at literature
    d. Thinking about your work
    e. Performing calculations to support your research
    f. Writing up your work

3. When taking your notes, how do you organise them? (indexing, creating sections etc)

4. Do you use any technology to aid with your note recording? (instruments: tablets, phones, cameras, recording equipment). Also prompt for inadvertent use of technology such as emailing yourself?

5. How do you link any digital resources or notes to paper based notes?

6. Where is your data / research output stored?

7. Are you concerned about IP?
    a. Do your records or notes need to be kept secure?
    b. Are there limits on who you can share your data with?
    c. Does your data need to be kept for a specific period of time?
    d. Does your data require any 3rd party sign off?

8. Who do you collaborate with for work, and who do you share your work with?
    a. Do you share your work for feedback?
    b. Is sharing your work useful?
    c. Are there people you **need** to be able to share your work with
    d. Before you share your work, do you write up your notes or change the format first?

9. Do you use reference management software, if so what?

10. Imagine you're trying to locate a piece of work or some notes from 6 months ago?
    a. How would you locate these notes
    b. How would you locate your data

11. Imagine that there is a fire in your lab and all of your paper notebooks are destroyed?
    a. How much of your work would be lost?
    b. How could you go about recovering this work?

12. If you fell under a bus tomorrow and were indisposed for a while, how would your supervisor/industry sponsors/colleagues access your work?

13. Where are all of your notes backed up, Electronic and Paper?

14. Have you used ELNs before?
    a. What did you like and didn’t you like?
    b. If you did use one, and you stopped using one, why did you stop?

15. What would you expect that an ELN would be able to do for you?

16. How could an ELN make recording your work better?

17. What equipment are you allowed to take into the lab?

18. Do you have any further comments on ELNs and Notetaking in the lab in general?
